# Trends in provision of photodynamic therapy and clinician attitudes: a tracker survey of a new health technology

**DOI:** 10.1186/1472-6963-5-34

**Published:** 2005-05-10

**Authors:** Robbie C Foy, Barny Foot, Jill Francis, Usha Chakravarthy, Richard PL Wormald

**Affiliations:** 1 The Centre for Health Services Research, University of Newcastle upon Tyne, UK; 2 The Royal College of Ophthalmologists, London, UK; 3 Health Services Research Unit, University of Aberdeen, UK; 4 Queen's University and Royal Victoria Hospitals, Belfast, UK; 5 Moorfields Eye Hospital, London, UK

## Abstract

**Background:**

There has been debate about the cost-effectiveness of photodynamic therapy (PDT), a treatment for neovascular age-related macular degeneration. We have been monitoring trends for the provision of PDT in the UK National Health Service. The fourth annual 'tracker' survey took place as definitive National Institute for Clinical Excellence (NICE) guidance was issued. We assessed trends in PDT provision up to the point of release of the NICE guidance and identified likely sources of pressure on ophthalmologists to provide PDT.

**Methods:**

National postal questionnaire survey of clinicians with potential responsibility for PDT provision. The survey explored reported local provision, beliefs about the effectiveness of PDT and what sources of opinion might influence attitudes towards providing PDT.

**Results:**

The response rate was 73% (111/150). Almost half of the surveyed ophthalmology units routinely provided PDT, as part of a trend of steady growth in provision. The proportion of respondents who believed that further proof of effectiveness was required has also declined despite the absence of any new substantial evidence. Attitudes towards providing PDT were positive, on average, and were more strongly associated with perceived social pressure from local colleagues than from other sources. Local colleagues were seen as being most approving of PDT.

**Conclusion:**

Those responsible for implementing the NICE guidance need to address ophthalmologists' beliefs about the evidence of effectiveness for PDT *and *draw upon supportive local individuals or networks to enhance the credibility of the guidance.

## Background

Age related macular degeneration (ARMD) is the commonest cause of severe loss of central vision in people aged over 50 in the Western world [1] and accounts for almost 50% of those registered as blind or partially sighted in the UK [2]. One form of the disease, neovascular ARMD, is more aggressive and accounts for up to 15% of cases [1]. Evidence from two randomised trials indicates that photodynamic therapy (PDT), administered five to six times over a two year period, reduces the relative risk of losing three or more lines of visual acuity over two years [3,4]. In particular, a subgroup analysis has shown a statistically significant benefit in the prevention of visual loss in people with wholly or predominantly classic choroidal neovascularisation (CNV). These findings contributed to pressure upon the UK National Health Service (NHS) from both patient and professional groups to make this therapy routinely available [5,6]. However, systematic reviews have highlighted reservations with respect to the reliance placed on the subgroup analysis [7-9]. Concern about the cost-effectiveness of PDT [10] prompted the National Institute for Clinical Excellence (NICE) to undertake a technology appraisal of PDT. Yet, during the protracted course of the technology appraisal and subsequent appeals, there was evidence that ophthalmology units were establishing services or referring patients for PDT.

When it was released in September 2003, the NICE guidance specified that only the subgroup of patients with 100% classic CNV should be eligible for PDT. Ideally, strategies to promote effective practice should be tailored according to identified local needs and barriers [11]. The rate of uptake of the NICE guidance may be influenced by several factors, such as ophthalmologists' perceptions of treatment benefit and time required to establish new services. Groups such as local colleagues or professional bodies may themselves influence clinicians' beliefs about treatment benefit. Hence the promotion of the NICE guidance may be more effective if it takes account of such professional norms.

We have been conducting a series of 'tracker' surveys to follow trends in the uptake of PDT. Our fourth survey, coincidently conducted as the final NICE guidance was being released [12], addressed whether provision of PDT had continued to expand despite the absence of the guidance and whether perceived professional norms represent a barrier to its implementation.

## Methods

Questionnaire design and administration were similar to those used in the previous three surveys [13-15]. We sought information about local treatment and referral policies for neovascular ARMD and the threshold of clinical benefit considered sufficient to justify the use of PDT. Benefit was rated on the basis of the number of patients that the respondent would be willing to treat to prevent the loss of three lines of visual acuity for two years for one patient at a given fixed cost of treatment of £8000 per patient (known as the number needed to treat (NNT)). This cost was estimated from the TAP study treatment protocol [1]. Six options for the NNTs were presented, four based upon the point estimate of effect and upper and lower limits of the 95% confidence intervals reported in the Cochrane Review [7], and two further categories of 1 in 50 and 1 in 100 for comparative purposes.

We used two constructs from the Theory of Planned Behaviour [16] to measure attitudes and subjective norms (which we shall now refer to as perceived social pressure) concerning the use of PDT. The questionnaire incorporated previously recommended scales and items to measure attitudes and perceived social pressure for a specific action: the treatment of patients with predominantly (more than 50%) classic sub-foveal CNV using photodynamic therapy [17]. Attitudes towards this use of PDT were measured using 1–7 Likert scales for four items (beneficial/harmful to patients; an excellent/poor use of resources; clinically/not clinically effective; good/bad practice). Ophthalmologists might feel under pressure from different groups. Four perceived social pressure items asked how much each of the following would approve or disapprove of the use of PDT: local colleagues, the Royal College of Ophthalmologists, NICE, and other ophthalmologists. Local colleagues might encompass both (mainly) ophthalmologists and others; whilst 'other ophthalmologists' would refer to any others encountered via formal and informal networks. The Royal College of Ophthalmologists and NICE refer to (perceived) official policy from each organisation.

Following pre-testing of the additional theory-based items, the questionnaire was posted to all clinical directors or lead consultants in NHS ophthalmology units within the UK in the autumn of 2003. The units were identified using the Royal College of Ophthalmologists' database. One reminder was sent to non-respondents.

Changes in service provision and beliefs were analysed using the χ^2^-test, and the normal z-test to compare two proportions. P values are presented. Associations between attitudes (the combined means of the four items) and each of the four perceived social pressure items were measured using multiple regression analysis. Differences between levels of perceived approval from the four potential sources of social pressure were tested using the repeated measures ANOVA.

## Results

Out of 150 questionnaires, 111 (73%) were returned completed, a slightly lower response rate than those for the previous three surveys (82%, 79% and 80% respectively). No variations in response rate were detected by health region either overall or in the individual surveys.

**Figure 1:**
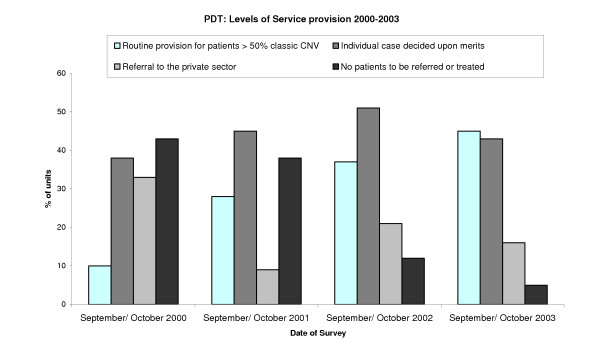
Trends in the reported provision of photodynamic therapy over 2000–2003.

The proportion of units reporting routine provision of PDT for patients with more than 50% classic sub-foveal CNV significantly increased over four years from 8.5% to 41% (p < 0.001), making this the most common reported policy for the first time. The proportion of units referring or treating no patients fell from 35% to 5% (Figure 1, p < 0.001).

There was a significant change in beliefs of what constituted a worthwhile clinical benefit over 2000–1 (p = 0.01), but no significant change was detected over 2001–2 (p = 0.97), or 2002–3 (p = 0.13) (Table 1) The proportion of respondents requiring further evidence before supporting the use of PDT fell from 33% to 11% over the four years. Lower thresholds supporting the use of PDT were associated with greater reported provision over the four surveys (χ^2 ^= 20.4, df = 8, p = 0.01; χ^2 ^= 25.2, df = 8, p = 0.003; χ^2 ^= 21.2, df = 8, p = 0.007, χ^2 ^= 17.0, df = 8, p = 0.03 respectively).

**Table 1:** Beliefs about clinical benefit and evidence of effectiveness over 2000–2003.

**Threshold of clinical benefit that would make offering PDT as a treatment worthwhile**		**Survey year**			
		
		**2000**	**2001**	**2002**	**2003**
To prevent the loss of 3 lines of visual acuity over 2 years	At least In 1 person for every 7 treated	22 (19%)	31 (27%)	30 (28%)	36 (37%)
	In 1 person for every 4 treated	35 (30%)	37 (32%)	38 (35%)	34 (35%)
	In 1 person for every 2 treated	21 (18%)	27 (23%)	20 (19%)	16 (17%)
Further evidence of effectiveness required		39 (33%)	20 (19%)	20 (17%)	11 (11%)
**Total**		117	108	115	97

The mean score for attitude was 4.86 (sd = 1.07), based on the 1–7 scale where higher scores represented more positive attitudes towards PDT for the treatment of patients with predominantly (more than 50%) classic sub-foveal CNV. Internal consistency was acceptable for the four attitude items (Cronbach's α = 0.69). The mean scores for perceived social pressure ranged from 2.86 for local colleagues to 3.82 for NICE, where lower scores represent greater levels of perceived approval (Table 2). A repeated measures ANOVA showed that perceived approval differed significantly as a function of the source of perceived social pressure (F(3,91) = 15.58, p < 0.001). NICE was seen as most disapproving of PDT use for the given indication; this was significantly different from scores for the other three sources of pressure (p < 0.001).

**Table 2:** Mean perceived social pressure for the treatment of patients with predominantly (more than 50%) classic sub-foveal CNV using photodynamic therapy where possible scores range from 1 (strongly approve) to 7 (strongly disapprove).

**Source of perceived social pressure**	**Mean**	**Standard deviation**
Local colleagues	2.86	1.34
Royal College of Ophthalmologists	3.01	1.23
National Institute for Clinical Excellence (NICE)	3.82	1.49
Other ophthalmologists	3.00	1.41

In the multiple regression analysis, scores for the four sources of social pressure significantly predicted attitude scores, R = 0.51, F(4,93) = 7.67, p < 0.001 (Table 3). Inspection of individual standardised regression coefficients (beta weights) showed that only perceived social pressure from local colleagues was a significant predictor of attitudes (p < 0.01).

**Table 3:** Multiple regression of scores for perceived social pressure on attitude scores. (Beta weights are negative because perceived social pressure and attitude scores were scored in opposite directions.)

Dependent variable	Independent variables	β	R	R^2^	Adjusted R^2^
Attitude concerning the use of PDT	Social pressure from local colleagues	-0.38**			
	Social pressure from the RCO	-0.03			
	Social pressure from NICE	-0.05			
	Social pressure from other ophthalmologists	-0.14			
			0.51***	0.26	0.22

## Discussion

By the time that the NICE guidance was released, almost half of the surveyed ophthalmology units were already routinely providing PDT. The proportion of respondents who believed that further proof of effectiveness was required has also declined despite the absence of any new substantial evidence. Attitudes towards providing PDT were positive, on average, and were more strongly associated with perceived social pressure from local colleagues than from other sources. Local colleagues were seen as being most approving of PDT.

There were several limitations to our methods. First, survey respondents may have held stronger views, or otherwise, about the merits of PDT compared with non-respondents. Second, additional variation in our findings over time may have been introduced by differences in respondents over each survey, although a sub-analysis provision of PDT for the units that responded to all four surveys (n= 76 (51%)) indicated similar findings as for the overall survey. Third, reported policies may differ from those used in practice. Fourth, the issue of NICE guidance around the time of the last survey may have altered responses. This seems unlikely to be a major factor, given the time taken to establish new services or referral policies. We also found no significant difference in attitudes towards PDT between those respondents who said they had read the NICE guidance and those who had not. Fifth, it would be wrong to assume the direction of causality was such that perceived social pressure influenced attitudes; more positive attitudes towards PDT might have preceded perceptions about which source approved or disapproved of PDT use. Finally, we enquired only about a limited number of sources of social pressure. Other sources might have included patients or the commercial sector, although social desirability bias might have led to an underestimation of effects of the latter.

In a classic case of 'technology creep' [18], PDT has become an established treatment in the NHS prior to national guidance and in the absence of new supporting evidence. This raises the question as to whether the routine application of new health technologies should be subject to a strict national moratorium. Proponents of PDT would argue that such a moratorium would stifle local service innovation, especially in light of the time taken to develop and issue final national guidance. However, we have previously suggested that the long time scale required to issue more controversial technology assessments may work in favour of the advocates of new technologies by forcing the hand of policy-makers [15].

There is also a risk of 'indication creep', whereby the use of an intervention expands beyond its recommended indication and results in less cost-effective use of health care resources. The NICE guidance specifies that only patients with 100% – as opposed to at least 50% – classic CNV should be eligible for PDT. The uptake of this guidance will partly depend upon the perceived credibility of the messenger. Our findings suggest that ophthalmologists are currently more likely to be influenced by local colleagues than by NICE, thereby posing a potential threat to consistent adherence to the guidance.

All of this means that those responsible for implementing the NICE guidance need to address ophthalmologists' beliefs about the evidence of effectiveness for PDT *and *draw upon supportive local individuals or networks to enhance the credibility of the guidance.

The implementation of national guidance requires monitoring. We plan to conduct one further survey to audit the reported uptake of the NICE guidance and measure the extent of any 'indication-creep'.

## Abreviations

PDT – Photodynamic Therapy

ARMD – Age related Macular degeneration

NICE – National Institute for Clinical Excellence

CNV – choroidal neovascularisation

NHS – UK National Health Service

## Authors' Contributions

RF participated in study design, interpreted the findings and wrote the first draft. BF conceived the idea for the study and conducted the analysis of trends. JF conducted the analysis and helped with the interpretation for the theory-based questions. All authors participated in the design of the study, were involved in interpretation of the findings, drafted or critically revised the article, and gave final approval of the version to be published.

## Competing interests

The author(s) declare that they have no competing interests.

## Pre-publication history

The pre-publication history for this paper can be accessed here:


